# Editorial: Skin lesion vitality assessment for forensic science: Current research and new perspectives

**DOI:** 10.3389/fmed.2022.969932

**Published:** 2022-08-15

**Authors:** Isabel Legaz, Vittorio Fineschi, Burkhard Madea, Stefano Bacci

**Affiliations:** ^1^Department of Legal and Forensic Medicine, Faculty of Medicine, Biomedical Research Institute (IMIB), Regional Campus of International Excellence “Campus Mare Nostrum”, University of Murcia, Murcia, Spain; ^2^Department of Anatomical, Histological, Forensic Medicine and Orthopedic Science, Faculty of Pharmacy and Medicine, Sapienza University of Rome, Rome, Italy; ^3^Institute of Legal Medicine, University Hospital Bonn, Bonn, Germany; ^4^Research Unit of Histology and Embriology, Department of Biology, University of Florence, Florence, Italy

**Keywords:** forensic sciences, forensic pathology, wound age estimation, lesion vitality, wound healing

Lesion vitality demonstration is one of the most challenging topics in forensic pathology (1–5). The demonstration of viability refers to determining whether an injury was caused ante- or post-mortem, so that it can be asserted with a high degree of probability that injuries objectified on a cadaver may be the basis of the cause of death (6–10). This specific field of research has been much studied over the years, and today the literature consists of a large body of in-depth studies that span three major strands. Quantitative analysis in biological fluids and tissues of various markers, immunohistochemistry, and ribonucleic acids studies particularly on epigenetics.

This editorial summarizes the contributions to the Frontiers Research Topic “*Skin Lesion Vitality Assessment for Forensic Science: Current Research and New Perspectives*” appearing in the Frontiers in Medicine section Translational Medicine. In particular, this Research Topic collects various contributions highlighting new types of analysis and methods to deal more efficiently with issues relating to the vitality of the lesion, a very important topic of forensic analysis (11–13). To better address and clarify the issues relating to this research field, the topic consists of a part dedicated to reviews on general or specific arguments in another containing research articles on specific topics.

## Review of general arguments

The first article in this collection by Collados Ros et al. reviews the forensic impact of the omics sciences involved in wounds to clarify different aspects of the diagnosis of vital injuries, time of appearance, age estimation, and the wound's vitality. Light is shed on the role of omics research during wounding, identifying different cytokines and other inflammatory mediators and cells involved in the specific stage of the wound healing process, showing great utility in estimating the age of a wound.

If this review addresses general issues relating to the vitality of the lesion, Maiese et al. review current knowledge about the vitality of ligature marks since this argument is a challenge for the forensic pathologist. The authors conclude that to ensure high reliability in court cases, forensic investigation in hanging should rely on modern and proven markers. Furthermore, given the difficulties of detecting vitality in a hanging groove, the authors recommend the use of various techniques using different markers and comparing the results obtained.

## Review of specific arguments

Concerning cellular tools in wound vitality, Ishida et al. review the role of bone marrow-derived cells as promising markers of potential usefulness in forensic applications. However, suggest the authors, since the purpose of wound age estimation is to present objective evidence in court when only a single marker is investigated, contradictory results are often obtained, eventually confusing the interpretation of data. Consequently, various populations of bone marrow-derived cells should be investigated during forensic analysis.

Examining the use of molecular tools in wound vitality, Prangenberg, Doberentz, Madea et al. reviewed the forensic value of aquaporins, their range of applications, current limits, and future implementations. The authors offer an overview of fields of application and applicable aquaporins as well as supplementary biomarkers. Thus, aquaporins are studied not only for skin injury, age of wound, and viability, but also for what emerges in literature on this subject concerning drowning, burns, trauma, suffocation/occlusion, intoxication, sudden cardiac death, and SIDS. A combination of immunohistochemistry and gene expression analysis seems useful to increase statistical significance in each case.

The same authors Prangenberg, Doberentz, Mawick, Madea et al. review the use of heat shock proteins in the practice of forensic analysis. These proteins act as molecular chaperones with cytoprotective functions that support cell survival under lethal conditions. Besides, they are expressed in specific cellular compartments and have many functions. In forensic analysis, results have indicated that Hsp70 is a helpful marker to estimate survival time. Therefore, this area of research appears to be potentially crucial in studies relating to lesion vitality.

De Simone et al. review the role of miRNAs as novel molecular biomarkers for dating the age of wound production. Some studies highlight whether the animal died during the day or at night, considering the modification of other miRNAs. miR-200c is a critical determinant that inhibits cell migration during skin repair after injury and may contribute to age-associated alterations in wound repair. miRNAs play an essential role in moderating cellular adaptations in drug abuse and addiction. For this reason, an exciting field of application of miRNAs is as an anti-doping marker.

## Research articles on specific topics

Bertozzi et al. investigated the possibility of using immunohistochemistry in the putrefied body. The results showed that most of the tested markers were highly expressed in putrefied skin for up to 15 days from death, leading to the conclusion that the use of various cellular markers allows only a qualitative evaluation of their expression, and consequently, other studies are necessary to deepen the issues considered.

Zhang et al., investigated the immunohistochemical expression of ubiquitin, which is involved in heat shock response and could be regarded as a valuable marker for diagnosing traces of antemortem compression in hanging furrows. The authors demonstrate that the depletion of ubiquitin expression revealed in neck compression may be caused by the impaired conversion of conjugated to free ubiquitin and failure of *de novo* ubiquitin synthesis. Therefore, the ubiquitin expression in the ligature mark can be considered a valuable marker for diagnosing traces of antemortem compression.

Gauchotte et al. evaluated the sensitivity and specificity of CD15 and myeloperoxidase (MPO), co-stained with glycophorin C, compared to standard histology, in a series of fresh medico-legal wounds and post-mortem controls in human experimental surgical model perspective. The detection of an inflammatory infiltrate based on histology is the gold standard, being highly specific but showing very low sensitivity in fresh wounds. The double staining for MPO or CD15/glycophorin C is a novel and interesting technique for detecting early vital reactions.

## Conclusions

The compilation of this Research Topic provides an occasion for the meeting of expert scientists, from different schools in the world, whose research is aimed toward lesion vitality. The published paper reviews are timely in clarity and exposition and the research articles propose new methods and fields of research that will certainly be deepened in the near future. In conclusion, from this collection emerges the idea that the issue of the vitality of the lesion is extremely significant, creating occasions to propose new molecular techniques to understand the role of the genome, proteome, and metabolome, adding to consolidated methods and opening new horizons to clarify different aspects of the diagnosis of vital injuries, time of appearance, age estimation, and the wound's vitality (14).

## Author contributions

All authors listed have made a substantial, direct, and intellectual contribution to the work and approved it for publication.

## Conflict of interest

The authors declare that the research was conducted in the absence of any commercial or financial relationships that could be construed as a potential conflict of interest.

## Publisher's note

All claims expressed in this article are solely those of the authors and do not necessarily represent those of their affiliated organizations, or those of the publisher, the editors and the reviewers. Any product that may be evaluated in this article, or claim that may be made by its manufacturer, is not guaranteed or endorsed by the publisher.
